# A new extract of the plant *calendula officinalis *produces a dual *in vitro *effect: cytotoxic anti-tumor activity and lymphocyte activation

**DOI:** 10.1186/1471-2407-6-119

**Published:** 2006-05-05

**Authors:** Eva Jiménez-Medina, Angel Garcia-Lora, Laura Paco, Ignacio Algarra, Antonia Collado, Federico Garrido

**Affiliations:** 1 Servicio de Análisis Clínicos e Inmunologia, Hospital Universitario Virgen de las Nieves, Universidad de Granada, Av. de las Fuerzas Armadas 2, 18014 Granada, Spain; 2 Departamento de Ciencias de la Salud, Universidad Jaén. Jaén, Spain; 3 Unidad de Investigación, Hospital Universitario Virgen de las Nieves, Granada, Spain

## Abstract

**Background:**

Phytopharmacological studies of different *Calendula *extracts have shown anti-inflamatory, anti-viral and anti-genotoxic properties of therapeutic interest. In this study, we evaluated the *in vitro *cytotoxic anti-tumor and immunomodulatory activities and *in vivo *anti-tumor effect of Laser Activated Calendula Extract (LACE), a novel extract of the plant *Calendula Officinalis (Asteraceae)*.

**Methods:**

An aqueous extract of *Calendula Officinalis *was obtained by a novel extraction method in order to measure its anti-tumor and immunomodulatory activities *in vitro*. Tumor cell lines derived from leukemias, melanomas, fibrosarcomas and cancers of breast, prostate, cervix, lung, pancreas and colorectal were used and tumor cell proliferation *in vitro *was measured by BrdU incorporation and viable cell count. Effect of LACE on human peripheral blood lymphocyte (PBL) proliferation *in vitro *was also analyzed. Studies of cell cycle and apoptosis were performed in LACE-treated cells. *In vivo *anti-tumor activity was evaluated in nude mice bearing subcutaneously human Ando-2 melanoma cells.

**Results:**

The LACE extract showed a potent *in vitro *inhibition of tumor cell proliferation when tested on a wide variety of human and murine tumor cell lines. The inhibition ranged from 70 to 100%. Mechanisms of inhibition were identified as cell cycle arrest in G0/G1 phase and Caspase-3-induced apoptosis. Interestingly, the same extract showed an opposite effect when tested on PBLs and NKL cell line, in which *in vitro *induction of proliferation and activation of these cells was observed. The intraperitoneal injection or oral administration of LACE extract in nude mice inhibits *in vivo *tumor growth of Ando-2 melanoma cells and prolongs the survival day of the mice.

**Conclusion:**

These results indicate that LACE aqueous extract has two complementary activities *in vitro *with potential anti-tumor therapeutic effect: cytotoxic tumor cell activity and lymphocyte activation. The LACE extract presented *in vivo *anti-tumoral activity in nude mice against tumor growth of Ando-2 melanoma cells.

## Background

Plants have a long history of use in the treatment of cancer. Active principles of *Angelica Gigas*, *Catharanthus roseus, Podophyllum peltatum, Podophyllum emodii, Taxus brevifolia, Ocrosia elliptica, and Campototheca acuminata *have been used in the treatment of advanced stages of various malignancies in the clinical setting [1,2]. Furthermore, many phytochemicals with different pharmacological properties have shown responses for the prevention or treatment of different tumors, e.g., flavones, flavanols, isoflavones, catechins, and taxanes [3-7]. Numerous drugs are used in cancer chemotherapy but most exhibit cell toxicity and can induce genotoxic, carcinogenic and teratogenic effects in non-tumor cells [8,9]. These side effects limit the use of chemotherapeutic agents despite their high efficacy in treating target malignant cells. Therefore, the search for alternative drugs that are both effective and non-toxic in the treatment of cancers is an important research line [10]. In fact, increased efforts are being made to isolate bioactive products from medicinal plants for their possible utility in cancer treatment [11].

Flowers of the plant *Calendula officinalis*, commonly known as "Marigold", are used in the West and in Asia for their anti-inflammatory properties [12,13].

Phytopharmacological studies of different *calendula *extracts have shown anti-viral activity, anti-HIV properties of therapeutic interest [14], and anti-genotoxic properties [15]. In clinical studies, *Calendula *was highly efficacious in the prevention of acute dermatitis in cancer patients undergoing postoperative irradiation [16]. Its cytotoxic effect on tumor cell lines in vitro and its anticancer efficacy *in vivo *was briefly outlined 20 yrs ago [17]. Chemical constituents of *C. Officinalis *include some triterpenes, triterpene oligoglycosides, and flavonol glycosides [18,19]. The aim of the present study was to evaluate the *in vitro *cytotoxic anti-tumor and immunomodulatory activities of a novel extract of the plant *Calendula Officinalis *(LACE: Laser Activated Calendula Extract). We show that LACE demonstrated a potent *in vitro *growth inhibition of several tumor cell lines, whereas it induced proliferation and activation of peripheral blood lymphocytes (PBLs). The mechanisms of this inhibition were identified as cell cycle arrest and apoptosis induction. Furthermore, the LACE extract presented anti-tumor activity *in vivo *in nude mice.

## Methods

### Preparation of the Calendula Extract (LACE)

The aqueous extract of *Calendula officinalis *(LACE) was obtained by subjecting the flower of this plant to the following patented process (patent n° EP 1 339 420 B1). First, the flowers were washed with water and comminuted by conventional methods. The flowers were treated with laser radiation at a wavelength of 650 nm for 15 min, followed by the suspension of 100 to 250 g of laser-treated plant in 1 L of water. The suspension was placed on a rocking platform at 4°C for 7–15 days and periodically treated with laser during this period. Then, the liquid phase was separated from the solid, an ochre-colored aqueous extract was obtained and stored in a freezer at -70°C. A second aqueous extract of *Calendula officinalis *was obtained following an identical process but without laser treatment (*calendula *extract, CE).

For the assays, the extract was concentrated to dryness in a lyophilizer, the dried preparation was homogenized in culture medium at concentration of 10 mg/ml (stock solution), and filtered through a 0.45 μM Millipore.

### Cell lines and cell culture

The following tumor cell lines were used: B16 murine melanoma, B9 murine MCA-induced fibrosarcoma, ANDO-2 human melanoma, MDA MB231 human breast cancer, AGS human gastric cancer, DU-145 human prostate cancer, A-549 human lung cancer, IMIN PC-1 human pancreatic cancer, DLD1 colon carcinoma, HeLa human cervical adenocarcinoma, U937 monocytic and Jurkat T lymphoma leukemias, and NKL, which was established from PBLs of a patient with LGL leukemia [20]. All cell lines were obtained from the American Type Culture Collection (Manassas, USA) except for the B9 cell line, which was generated at our laboratory, and the Ando 2, IMIM PC-1, and NKL cell lines, kindly provided by P. Coulie (Unite de Genetique Cellulaire, Louvain University, Brussels, Belgium), F. X. Real (Instituto Municipal de Investigaciones Medicas, Barcelona, Spain), and Dr. M. Lopet-Botet (Universidad Pompeu-Fabra, Barcelona, Spain), respectively. Cell lines derived from solid tumors were grown at 37°C in a humidified atmosphere of 5% CO_2 _in DMEM culture medium (Gibco, Paisley UK) supplemented with 10% heat-inactivated fetal bovine serum (Life Technologics, Milan Italy), antibiotics, and glutamine. U937 and JURKAT leukemia cells were cultured in RPMI. The NKL cell line was cultured in RPMI 1640 with 10% heat-inactivated human AB serum (Sigma Chemical, St Louis, MO; USA) and human recombinant IL-2 (1000 U/ml; purity>97%, specific activity, 2 × 10^6 ^U/mg) (Roche, Nutley, NJ; USA).

### Lymphocyte proliferation assay

Human lymphocytes were isolated from venous blood by Ficoll-Hystopaque separation method. Proliferation of PBLs was analyzed *in vitro *using 5-bromo-2'-deoxyuridine (BrdU) labeling of DNA-synthesizing cells with the Brdu colorimetric ELISA Cell Proliferation kit, (Roche Diagnostics). PBLs were seeded in 96 well-microculture plates at a cell density of 5 × 10^4 ^per well. A dose/response curve was performed using different concentrations of LACE or CE (2 mg/ml to 15 μg/ml). Concanavalin A (5 μg/ml, Sigma) and IL-2 were used as positive control. After 48 h of culture in presence or absence of LACE, BrdU labeling reagent (final concentration 10 μM) was added and the cells were cultured during 24 h, then cells were fixed for 30 min and incubated with anti-BrdU for 1 h at 37°C. 100 μl of tetramethyl-benzidine (TMB) was used as substrate. Optical densities were determined at 370 nm using an ELISA microplate reader (Biotek, Power-Wave XS). Controls were: culture medium, cells cultured only in medium and cells incubated with anti-BrdU in absence of BrdU. All experiments were plated in triplicate wells and were performed at least three times.

### In vitro cytotoxicity assays

The effect of LACE or CE on tumor cell proliferation was assessed by measuring BrdU incorporation with the kit described above. Cells were plated in 96-well microculture plates (5 × 10^3 ^cells/well). After 24 h of culture at 37°C, a dose/response curve was performed as described above. Every 48 h, the culture medium was replaced and LACE or CE was added. After 48–96 h BrdU labeling reagent was added and cultured for a further 1–3 h. Assays were also performed by counting viable cells using Trypan Blue exclusion. Briefly, cancer cell lines were seeded into culture tissue-flask (1.5–2 × 10^5^/culture tissue-flask) or 6-well plates (NKL cells) and incubated for 24 h at 37°C in a humidified atmosphere of 5% CO_2_. Cells were then treated with 250 μg/ml of LACE in the culture medium, which was replaced every 48 h. After 4–6 days, the cells were collected by centrifugation and a small sample of the cell suspension was diluted in 0.4% Trypan Blue and cells were counted in a hemocytometer chamber. Each cell sample was counted in this way at least three times and each assay was repeated at least three times.

### Cell cycle distribution analysis

Briefly, cells were plated in six well plates (5 × 10^5 ^per well) or in culture tissue-flask (15 × 10^5^) and exposed continuously for 4 days to 250 μg/ml of LACE. The rate of DNA synthesis was examined by a BrdU incorporation method using FITC BrdU Flow Kit (BD Pharmingen) according to manufacturer's instructions. BrdU was then detected by using a method with DNasa cell treatment and FITC-conjugated anti-BrdU antibody. Cells were washed with 1 ml of 1 × BD Perm/Wash Buffer and 20 μl of 7-amino-actinomycin D was added. Analysis was performed with 50000 cells using Cell Quest Software and FACScan flow cytometer (Becton-Dickinson).

### Analysis of expression of cyclins and cyclin-dependent kinases (CDKs)

The cell lines AGS and JURKAT were exposed to 250 μg/ml LACE during 96–144 hours. The cells were washed with PBS and permeabilized with Citofix/Citoperm 30 min to 4°C, incubated with anti-cyclin D1 and E antibodies (BD Pharmigen) and finally propidium iodide was added. The cells were analyzed by inmunofluorescence as mentioned above.

Western blot analysis was performed for the analysis de CDK (CDK1/Cdc2, CDK2, CDK4, CDK6) and cyclins (A, B y D3). The Kit TransFactor Extraction (BD Biosciences) was utilized to extract whole proteins. Protein samples were separated by SDS/PAGE (10%) and then electroblotted onto a polyvinylidene difluoride membrane (Kit, Mini Trans-Blot Electrophoretic Transfer Cell; Bio-Rad). The membranes were saturated with 5% nonfat dry milk in TBST (25 mM Tris HCl, 140 mM NaCl and 0.1% Tween 20) and then incubated with anti-CDK1/Cdc2, CDK2, CDK4, CDK6, cyclin A, cyclin B and cyclin D3 antibodies. The goat anti-Mouse IgG-HRP (BD Biosciences Pharmingen) was used as the second antibody. The membranes were washed thoroughly with TBST and incubated for 5 to 30 min with colorimetric solution, Opti-4CN (Bio-Rad, Madrid, Spain).

### Annexin V binding assay to detect apoptotic cells

After treatment of cancer cells with LACE for four days, cells were detached from the culture tissue-flask with PBS containing 3 mM EDTA. These cells were then collected together with floating cells, washed twice with cold PBS, and resuspended in binding buffer at a concentration of 1 × 10^6^cells per ml. 100 μl of solution was incubated for 30 min at 4°C with 5 μl of Annexin V-PE antibody (BD Biosciences), and 5 μl of 7-amino-actynomycin D was then added. Cells were incubated for 15 min in darkness, and 400 μl of staining buffer was added before flow cytometry analysis.

### Assay for active caspase-3 expression

FITC conjugated monoclonal anti-active-caspase-3 antibody (BD Biosciences) was used to determine whether the protease Caspase-3 was involved in apoptosis induced by LACE. After treatment with LACE for 4 days, the cancer cells were washed twice with cold PBS and fixed and permeabilized in Cytofix/Cytoperm buffer. Then, cells were incubated with FITC-conjugated monoclonal rabbit anti-active human-caspase-3 antibody for 30 min. Cells were washed twice and 500 μl of 1 × Perm Wash Buffer was added before analysis by flow cytometry.

### In vivo toxicity assays

For *in vivo *studies, immunocompetent 6- to 8-week-old Balb/c, C57/BL6 and CBA mice and 3-month-old Wistar rats were obtained from the Animal Centre of our institution. Mean weight of mice and rats was 20 g and 150 g, respectively. Animals were housed in wire-topped plastic boxes kept on a 12 hour light/12 hour dark cycle under pathogen-free conditions. All studies of the animals were performed according to guidelines approved by our institution. Animals were randomly divided into groups of 10 mice or rats. LACE extract (0.2 ml) was administered orally by cannula daily for 5 weeks and the control group was similarly treated with 0.2 ml water. LACE concentrations used were: 11 mg/kg/day, 55 mg/kg/day, 550 mg/kg/day, and 2750 mg/kg/day. After 4 weeks of treatment, the animals were maintained for 4 weeks under standard conditions and assessed daily for systemic (listlessness, weight loss) or local (alopecia, skin reaction and leg motility) toxicity.

### Effect of LACE on solid tumor growth in nude mice

Athymic nude mice 6 weeks were obtained from Charles River (CRL, Barcelona) and all studies of the animals were performed according to guidelines approved by our institution. The human melanoma cell line ANDO-2 (5 × 10^6 ^cells) was injected subcutaneously at the back foot pad of mice. When tumors became visible about one week after injection, the animals were randomized into six groups: LACE-oral treated (50 mg/Kg of weight), LACE-intraperitoneal treated (25 mg/Kg of weight), taxol-intraperitoneal treated (5 mg/Kg of weight), control, controls treated with saline solution by oral or intraperitoneal route. LACE was administered orally three times a week during 12 weeks, LACE intraperitoneal route twice a week during 9 weeks and taxol was administered intraperitoneally twice a week during three weeks. All products were firstly administered in day 8 after cells injection. Tumor growth (or the long tumor diameter) was monitored with calipers three times a week measuring long tumor diameter. Results are expressed as mean size of tumors from each group ± SD. Each group was composed of 10 mice and the assays were performed at least three times. A comparison of the survival percentage between treated and control group was performed.

## Results

### LACE extract exerts mitogenic activity on PBLs

**Figure 1:**
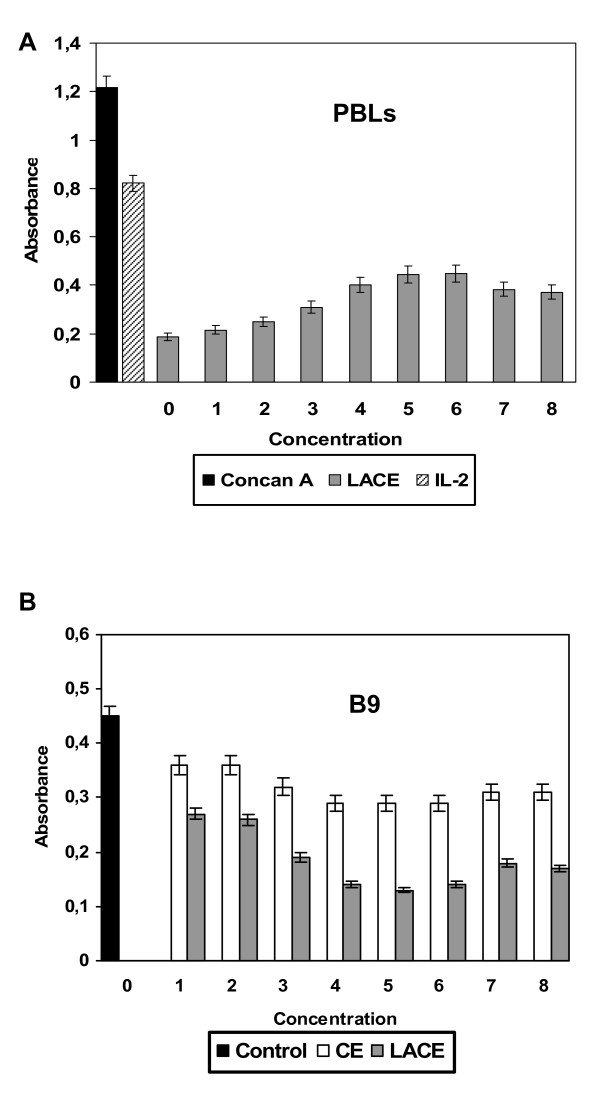
**Dose-response effect of LACE extract in PBLs and B9 fibrosarcoma murine cell line**. **a) **PBLs were cultured in medium alone or with various concentrations of LACE extract from 2 mg/ml (conc. 8) to 15.62 μg/ml (conc. 1) for 72 h. **b) **For B9 tumor cells were cultured with culture medium alone or various concentrations of LACE or CE for 72 h. Each column represents the mean of five independent experiments ± SD.

A complete dose-response study was performed to determine the action of LACE or CE on human PBLs, plating 5 × 10^4 ^cells in a 96-well tissue plate for 48–72 h with one of 8 concentrations of extract as follows: concentration n° 8 = 2 mg/ml, concentration n° 7 = half of concentration n° 8, and so on successively to a concentration n° 1 of 15.62 μg/ml, with concentration n° 0 representing cells cultured in medium alone. Fig. 1a depicts the absorbances when PBLs were incubated with LACE extract, showing a significant increase in proliferation of PBLs at extract concentrations of 125–500 μg/ml. The proliferation induced by LACE was 3 to 5-fold greater than in the control, reaching 30% of the level of proliferation induced by Concanavalin A and 50% of the proliferation induced by IL-2 (Fig. 1a). If the results of PBLs proliferation are expressed as Stimulation index (SI: absorbance of treated lymphocytes divided by absorbance control or unstimulated lymphocytes): SI_LACE _= 2.5, SI_IL2 _= 4.4, SI_conA _= 6.5.

Interestingly, LACE induced proliferation without previous stimulation of PBLs. When these assays were performed using the non laser-treated *calendula *extract, CE, the absorbance results were practically identical, indicating that both extracts produce a similar increase in PBL proliferation (data not shown). The principal chemical components of the aqueous extracts of *Calendula Officinalis *are: polysaccharides, proteins, fatty acids, carotenoids, flavonoids, triterpenoids and saponins (data not shown).

### LACE extract inhibits *in vitro *tumor cell proliferation

Exponentially growing cell lines (2.5–5 × 10^3 ^cells) were exposed to increasing concentrations of LACE or CE for 48–72 h in 96-well tissue plates as described above, and a dose/response curve was performed. Figure 1b depicts absorbance results when B9 murine fibrosarcoma cells were cultured with LACE extract. These results showed a significant decrease in absorbance, mainly in the 125 – 500 μg/ml concentration range, with an IC_50 _concentration of 60 μg/ml. Similar results were found for HELA and Ando 2 cell lines (data not shown). When these assays were performed with the non laser-treated CE extract, the decrease in absorbance was notably smaller (Fig, 1b). These results indicate that CE extract produced significantly less inhibition of tumor cell proliferation in comparison with LACE extract.

**Figure 2:**
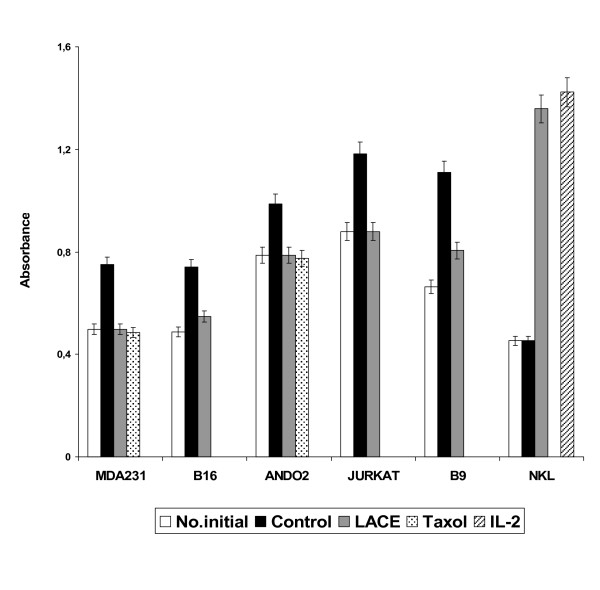
**Effect of LACE in proliferation of different cancer cell lines**. The MDA MB231, B16, ANDO-2, JURKAT, B9 and NKL tumor cell lines were treated or not with 250 μg/ml of LACE for 72–96 h. MDA MB231 and ANDO-2 were also treated with Taxol. Each column represents the mean of five independent experiments ± SD.

Following these screening assays, LACE extract was used in further experiments at a concentration of 250 μg/ml. Similarly results were obtained when other studied tumor cell lines were cultured with 250 μg/ml of LACE (a decrease in absorbance was observed with respect to untreated cells) (Fig. 2). Paclitaxel (Taxol) was used as control in these experiments, yielding similar results to those obtained with LACE (Fig. 2). The NKL leukemia cell line was the exception, since treatment with LACE extract induced an increase in proliferation. It should be taken into account that NKL cells are dependent on IL-2 for their growth and LACE and IL 2 induce similar levels of proliferation.

The correlation between optical densities and number of viable cells was analyzed, culturing 1–2 × 10^5 ^tumor cells in tissue-flask in medium alone (control) or with 250 μg/ml of LACE for 4–6 days. As shown in Table 1, there was a significant decrease in the final number of viable cells, with a growth inhibition of 70–100% versus control cells, except for the treated leukemia cell line U937, which showed a growth inhibition of 21%. Under an inverted phase contrast microscope, LACE-treated tumor cells showed morphological changes: rounded and granulated morphology, some vacuoles coming from cytoplasm, cell shrinkage and detached from culture plates of a high number of cells. These morphological changes were not observed in lymphocytes treated with LACE.

### Cell cycle phase distribution analysis of treated cells

**Figure 3:**
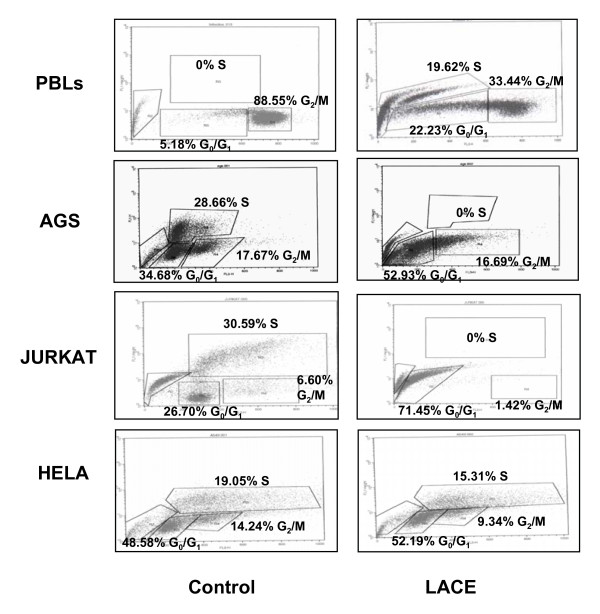
**Cell cycle analysis of cells treated with LACE**. PBLs were treated with LACE for 96 h, and cells were analyzed by flow cytometry. Similarly, tumor cell lines were treated with LACE for 96 h. Data indicate the percentage of cells in each phase of cell cycle. Results are representative of three independent experiments.

Cells were found accumulated in G_2_/M phase (88.55%) when PBLs were cultured in medium alone. However, when PBLs were cultured with addition of 250 μg/ml LACE, cells re-entered cell cycle, appearing in S phase (Fig. 3). In contrast, when a tumor cell line, e.g., AGS, was cultured in medium alone, cells were in cell cycle (34.68% G_0_/G_1_, 28.66% S, and 17.67% G_2_/M), and when 250 μg/ml LACE extract was added, cells showed a significant accumulation in G_0_/G_1 _phase (52.93% of cells) at the expense of the S phase population (Fig. 3). Similar results were found in Jurkat cells, where the percentage of LACE-treated cells in G_0_/G_1 _phase was 71.45% (Fig. 3). However, in other tumor cell lines, e.g., HELA cells, a slowing rather than an arrest of cell cycle was observed, with a partial accumulation of the cells in G_0 _/G_1 _phase (Fig. 3). Fractions of cells in the studied tumor cell lines are listed in the Table 2.

### LACE inhibits expression levels of CDKs- and cyclins-associated to G_1 _phase

**Figure 4:**
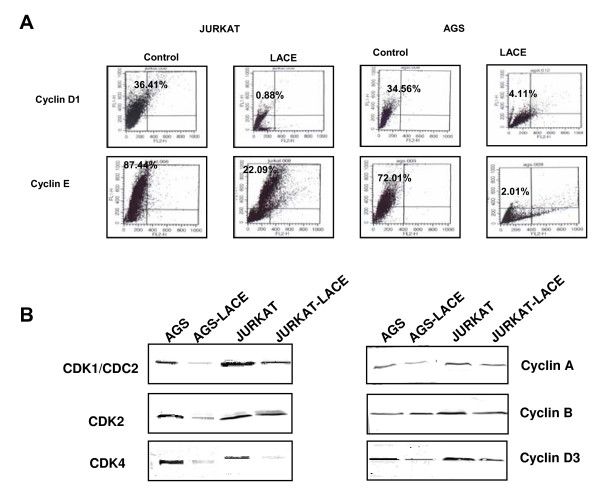
**Effect of LACE on cyclins and CDKs**. The cell lines AGS and JURKAT were cultured in alone medium or treated with LACE. **a) **Flow cytometric analysis was performed for the expression of cyclins D1 and E. The LACE extract produced a strong inhibition of expression of both cyclins. **b) **The western blot analyses showed that LACE produced down-regulation of cyclins D3 y A and CDK1/Cdc2, CDK2, CDK4, CDK6. The cyclin B expression was not modified.

We have found G_1 _cell cycle arrest in cells treated with LACE. The cyclins D1 and E are important regulator of progression through of G_1 _phase and entry into S phase. The AGS and JURKAT cells were treated with LACE during 96–144 hours. After this period, a decrease in the expression of these proteins was detected by immunofluorescence (Fig. 4a). The JURKAT cells cultured in alone medium present an expression of cyclin D1 and E of 36.41% and 87.44%, respectively; when these cells were cultured with LACE the cyclin D1 expression decrease to 0.88% and the cyclin E expression to 22.09% (Fig. 4a). Similar results were found in AGS tumor cell line, so the cyclin D1 expression decreases of 34.56% to 4.11% and the cyclin E expression decreases of 72.01% to 2.01% (Fig. 4a).

The western blot analyses of total cell lysates showed a decrease of protein levels of CDK1/Cdc2, CDK2, CDK4, CDK6, cyclin A and cyclin D3, in LACE-treated cells (Fig. 4b). The cyclin B expression was no altered in response to LACE (Fig. 4b).

### LACE induces apoptosis in tumor cells

**Figure 5:**
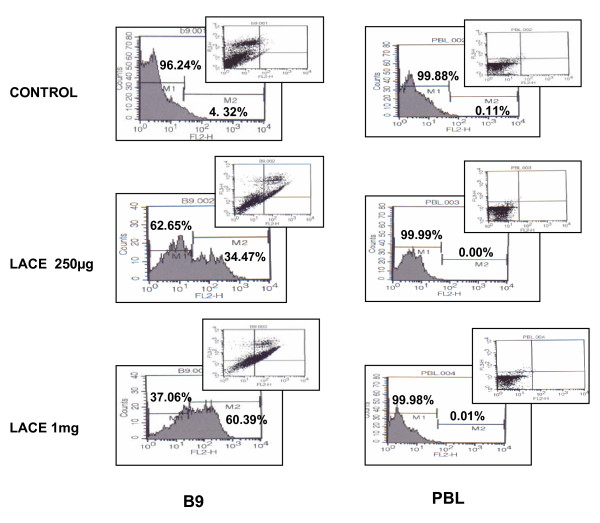
**Apoptosis induced by LACE**. B9 cell line was untreated or treated with 250 μg/ml or 1 mg/ml of LACE for 96 h. Cells were double-stained with annexin V and 7AAD and analyzed by flow cytometry. PBLs were also analyzed and LACE extract did not produce apoptosis. All experiments were performed at least three times and gave similar results.

Treatment of cancer cells with LACE led to the generation of a sub-G_1 _population suggestive of apoptosis. To quantitatively examine the ability of LACE to induce apoptosis, cancer cells were treated with 250 and 1000 μg/ml for 4 days. Cancer cells that were untreated or treated for 4 days were incubated with Annexin V-PE antibody in a buffer containing 7-amino-actinomycin D and analyzed by flow cytometry. Apoptosis of B9 cells increased from 4.32% in cells cultured in medium alone to 34.47% in those cultured with 250 μg/ml of LACE, and to 60.39% in those cultured with 1 mg/ml of LACE (Fig. 5). Similar results were found in the other tumor cell lines (Table 3). In contrast, LACE did not produce apoptosis in PBLs (Fig. 5).

### Expression of active human caspase-3

**Figure 6:**
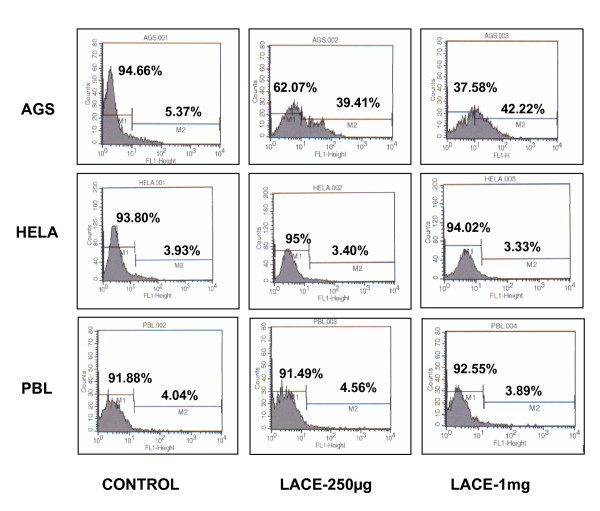
**Flow cytometric analysis of apoptotic populations using anti-active caspase 3 antibody**. AGS and HELA tumor cell lines were treated with 250 μg/ml or 1 mg/ml of LACE. Data indicate the percentage of cells positive for presence of active-caspase-3. Results are representative of three experiments

Since caspases are the main enzymes involved in the apoptotic pathway, it was investigated whether active caspase-3 was involved in LACE-induced apoptosis. Thus, the cancer cell lines were treated with LACE for 4 days, and cells were then permeabilized, fixed, and stained for active human caspase-3 and analyzed by flow cytometry. In the case of AGS cell line, the results clearly illustrated that untreated cells were primarily negative for presence of active-caspase-3, whereas around 40% of LACE-treated cells showed detectable active caspase-3 (Fig. 6). Expression of active caspase-3 was also detected in other tumor cell lines, whereas some lines, e.g., HELA, showed no caspase-3 expression after LACE treatment (Fig. 6). These results indicate that apoptosis in HELA cells was not induced by caspase-3. In PBLs, as expected, LACE treatment did not induce caspase-3 expression (Fig. 6).

### PBL subpopulations activated by LACE

**Figure 7:**
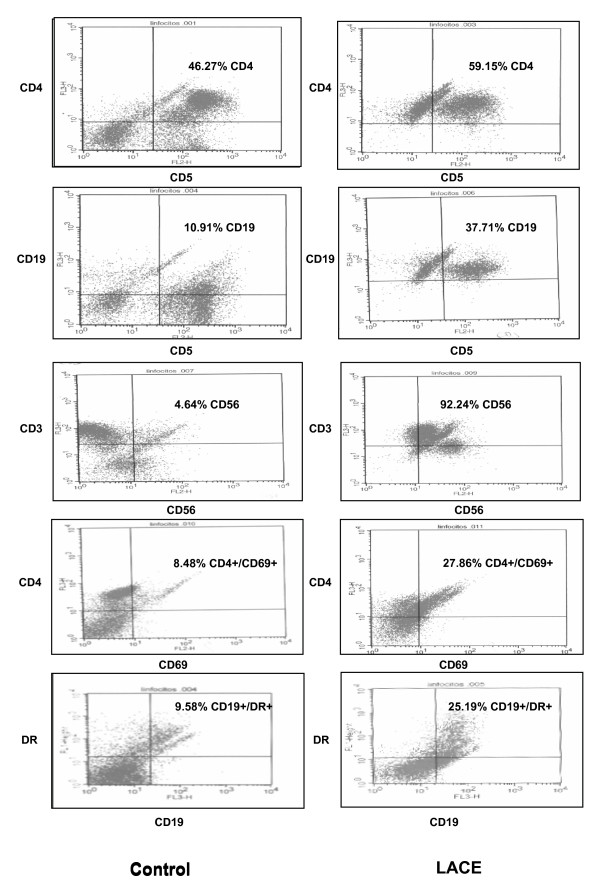
**Subpopulation analysis in PBLs treated with LACE**. PBLs treated or not with LACE were analyzed and results indicate an increase in CD4, CD19, and CD3-CD16-CD56 positive cells. This experiment was repeated at least three times and gave similar results.

We also analyzed the PBL subpopulations that proliferated after LACE treatment. Figure 6 depicts representative results of PBL subpopulations treated with 250 μg/ml LACE for 72–96 h. In control PBLs, the subpopulations were 46.27% CD4+, 10.91% CD19+ and 4.94% CD56+/CD3+. After LACE treatment, these populations significantly increased to 59.15% CD4+, 37.71% CD19+ and 92.24% CD56+/CD3+ (Fig. 7). Therefore, cells in proliferation were mainly B lymphocytes, CD4+ T lymphocytes, and NKT lymphocytes. Activated CD markers of lymphocytes (*e.g*., HLA-DR and CD69) were expressed by the lymphocyte subpopulations that proliferated with LACE treatment (Fig. 7), indicating that these lymphocytes were activated.

### Toxicity of LACE extract in mice and rats

After the above *in vitro *activities of LACE were established, investigation of its *in vivo *toxicity was the next step. In a preliminary study, Balb/c, C57/BL6, and CBA mice were orally administered with 11 or 55 mg/kg body weight of LACE daily for 30 consecutive days and no deaths were observed during this period. No differences in systemic or local toxicity were observed between LACE-treated groups and controls, although a dose increase to 550 mg/kg body weight resulted in a 50% reduction in the 15-day survival of these mice (LD_50_) (data not shown). At autopsy, the animals exhibited hepatic toxicity. LACE was also orally administered to rats at a dose of 11, 55 and 550 mg/kg body weight daily for 30 consecutive days, and no deaths or increases in systemic or local toxicity were observed. However, when the daily dose was 2750 mg/kg body weight, 50% of rats died by day 15 (LD_50_) (data not shown). After 30 days of treatment, surviving mice and rats were maintained under standard conditions and their mortality was recorded at 90 days post treatment. During this period, no toxic side effects, e.g., debility, loss of body weight or death, were observed.

### *In vivo *anti-tumor effect of LACE

**Figure 8:**
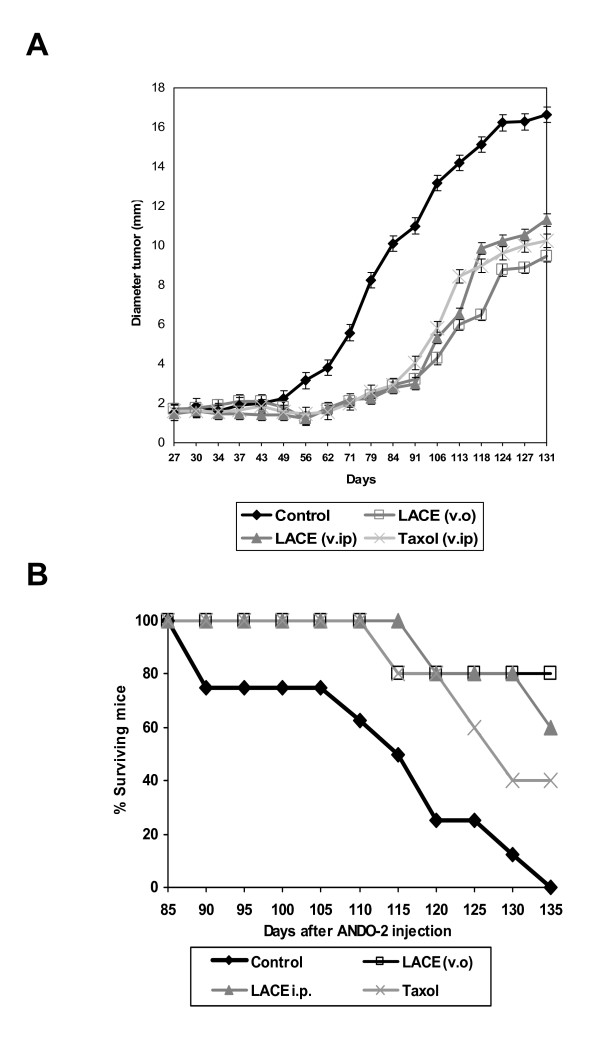
**Effect of LACE on the growth of ANDO-2 in nude mice**. **a) **There was not differences between the different control groups, while LACE induced tumor growth inhibition reached to about 60%. The Y-axis represents long tumor diameter and the X-axis represents time (day) after ANDO-2 cell injection. Each data point represents the mean ± SD of three independent experiments. **b) **Effect of LACE in survival time. The percentage survival was calculated as: % survival = number of survival mice in the test group/number of mice in the group. Data are representative of three independent experiments.

We have established in our laboratory a solid tumor model with nude mice bearing ANDO-2 human melanoma cell line. We next investigated the effect of LACE on this tumor model. The mice administered with LACE, oral (50 mg/kg of weight) or intraperitoneal (25 mg/kg of weight) route, showed similar tumor growth inhibition reached to about 60% (Fig. 8a). Similar results were found with Taxol (Fig. 8a). There was not differences between the different control groups. The untreated mice showed a fast progressive increase in tumor volume on the day 49 while in groups treated with LACE o Taxol occurred later on day 90 (Fig. 8a).

On the other hand, the survival of mice in LACE-treated groups was monitored and compared to control groups. At the end of assays, the survival was: 0% in control groups, 75% in LACE-oral group, 60% in LACE-intraperitoneal group and 40% in taxol group (Fig. 8b).

## Discussion

The main finding of the present study is the *in vitro *anticancer efficacy of a novel extract obtained from *Calendula Officinalis *against various cancer cell lines derived from human or murine solid tumors of different etiologies. In some cases, this extract, LACE, achieved 100% inhibition of growth (Fig. 2, Table 1). Importantly, this inhibition effect is exerted on tumor cells from different solid tumors and is not specific to a single tumor cell tissue. The *in vitro *growth inhibition of LACE extract was similar to that reported for Taxol in tumor cell lines [21], as shown in Figure 2.

The principal chemical components of the aqueous extracts of *Calendula Officinalis *are: polysaccharides, proteins, fatty acids, carotenoids, flavonoids, triterpenoids and saponins. The carotenoids components may be excited by visible radiation. Laser treatment of the *calendula *extract is necessary to detect this biological activity. A similar *calendula *extract without laser treatment, CE, produced only a slight inhibition of tumor cell growth (Fig. 1b). The increase in this biological activity is due to treatment with laser radiation, which may induce conformational changes, excitation or degradation of different molecules of the CE extract.

Studies performed to assess the mechanisms involved in the above *in vitro *effects showed that LACE induced cell cycle arrest in G_0_/G_1 _(Fig. 3 and Table 2). Furthermore, mechanistic investigation showed that LACE-induced G_1 _arrest mainly mediated via a down-regulation of cyclins D1, D3, E y A, and CDK1-Cdc2, CDK2, CDK4 and CDK6 (Fig. 4). These novel data may have clinical relevance, since most human malignancies exhibit aberrations in cell cycle regulation [22]. In fact, most anticancer agents derived from plants exert their effect via apoptosis induction in cancer cells [23-25]. The present data demonstrated that LACE induces apoptotic death and that the rate of apoptosis increases with higher LACE concentrations (Fig. 5, Table 3). The induction of apoptosis involved caspase-3-dependent mechanisms in some but not all tumor cell lines (Fig. 6), suggesting differential molecular determinants of apoptosis induction in different tumor cell lines.

In leukemia cell lines, LACE treatment inhibited 100% of growth in Jurkat cells and only 20% of growth in U937 cells, which may be explained by the different origin of the cells (lymphoma and monocytic, respectively) and/or the much higher growth rate of U937 cells, which double in number in less than 24 h. Interestingly, LACE induced proliferation of NKL leukemia cells (Fig. 2). The NKL cell line is dependent on IL-2 for its growth *in vitro *[20], indicating that it is in arrest phase of the cell cycle. Treatment with IL-2 or LACE produces cell cycle re-entry and similar proliferation values in NKL cells (Fig. 2). Likewise, a compound isolated from the fungus *Coriolus Versicolor*, Protein-bound polysaccharide K (PSK), also induces proliferation of NKL cells in absence of IL-2 [26]. PSK has shown anticancer activity *in vitro *in experimental models and in human clinical trials [27,28]. These anti-tumor activities can be largely attributed to activation of NK cells [29,30].

The second novel finding of this study is that LACE treatment induces proliferation and activation of human PBLs (Fig. 1 and Fig. 7). The CE extract, without laser treatment, also produces a similar increase in PBL proliferation. PBLs, which do not proliferate *in vitro *and are found in G_2_/M arrest, re-entered cell cycle with LACE treatment (Fig. 3).

PBL subpopulations in LACE-induced proliferation were CD4+, CD19+, and mainly CD3+/CD16/56+ (Fig. 7). The latter correspond to NKT cells, which have been shown to recruit and promote a response by downstream effectors in an IFN-γ-dependent manner, activating both NK and CTL anti-tumor activity [31,32]. These data, considered alongside the cytotoxic activity of LACE extract in tumor cell lines, indicate that it might have anticancer properties *in vivo*. In fact, preliminary results from our laboratory indicate that LACE can inhibit the growth of mouse tumor cells *in vivo *(unpublished results). Results obtained with PBLs and NKL cells indicate that cells in cell cycle arrest re-enter cell cycle with LACE treatment. In contrast, LACE treatment produces phase G_1 _cell cycle arrest in tumor cells in cell cycle (Fig. 3). Further research is required to determine whether a single active principle is responsible for this dual *in vitro *activity. Purification of LACE extract is in progress to identify its active principles. A further finding of this study was the low *in vivo *toxicity of this extract.

The LD_50 _for orally administered LACE was 550 mg/kg body weight in mice and 2750 mg/kg body weight in rats. Notably, the antiproliferative activity of LACE was not accompanied by systemic toxicity in mice or rats at a dose of 50 mg/kg body weight. The LACE extract showed *in vivo *anti-tumor activity in nude mice, the growth of Ando-2 tumor was reduced in a 60% (Fig 8). The anti-tumor efficiency of LACE was similar to obtained with other commonly used chemotherapy drug, paclitaxel. Furthermore, LACE produced higher prolongation of lifespan in tumor-bearing mice (Fig. 8).

The results of the present study are encouraging because LACE has shown significant inhibition of tumor growth *in vitro *and *in vivo*, therefore LACE or some components might be a promising chemotherapy candidate in treating cancers in clinic. Further experiments will focus on purification studies and the *in vivo *efficacy of LACE in other experimental mouse cancer models.

## Conclusion

LACE extract have demonstrated *in vitro *growth inhibition of various tumor cell lines. This is due to induction of cell cycle arrest and apoptosis. In contrast, it induced proliferation and activation of PBL cells. In addition, LACE present anti-tumor activity *in vivo *in nude mice.

## Abbreviations

LACE, Laser Activated Calendula Extract; CE, Calendula extract; PBLs, Peripheral Blood Lymphocytes; LGL, Large Granular Lymphocyte; TMB, Tetramethyl-benzidine.

## Competing interests

The authors declare that they have no competing interest. Materials for these studies were partially supported by a grant from Bomsund S.L. Malaga, Spain. The extraction process is patented by Bomsund S.L. The authors have no financial or non-financial relationship with the company Bomsund S.L. The authors have no interest, arrangement or affiliation with any product or organization that could be perceived as a real or apparent conflict of interest in the context of this manuscript.

## Authors' contributions

EJM and AGL performed the assays. IA and AC helped in some experiments. AGL and FG designed the study and drafted the manuscript. All authors have read and approved the final manuscript.

## Pre-publication history

The pre-publication history for this paper can be accessed here:



## Figures and Tables

**Table 1:** Effect of LACE on growth inhibition in tumor cell lines.

	**Cell N_o _. (×10^4^)**			
	**Initial**	**Control**	**LACE**	**Inhibition**
B16	25	440	120	76%
B9	12	120	29	72%
MDA MB231	20	50	20	100%
ANDO 2	20	32	20	100%
AGS	15	36	15	100%
DU 145	15	40	22	72%
HELA	15	25	16	90%
IMIN PC1	15	35	15	100%
A549	16	56	16	100%
DLD1	15	104	30	83%
U937	10	94	78	21%
JURKAT	20	34	20	100%

These results are representative of three independent experiments.

**Table 2:** Fractions of cells in specific phases of the cell cycle after culture of cancer cell lines in presence or absence of LACE.

	**DEAD**	**G2**	**S**	**G1**
AGS control	5.77%	17.67%	28.66%	34.68%
AGS LACE	12.24%	16.69%	0%	52.93%
A549 control	9.35%	13.43%	32.13%	29.55%
A549 LACE	8.94%	12.03%	3.40%	44.46%
Jurkat control	22.45%	6.60%	30.59%	26.70%
Jurkat LACE	11.61%	1.42%	0%	71.45%
HELA control	6.59%	14.24%	19.05%	48.58%
HELA LACE	14.77%	9.34%	15.31%	52.19%
B16 control	18.42%	6.13%	27.57%	33.84%
B16 LACE	10.98%	6.21%	4.57%	57.46%

Results are for one typical experiment out of three performed.

**Table 3:** Apoptosis induction in cancer cell lines after treatment with LACE for 4 days.

	**Control**	**LACE 250 μg/ml**	**LACE 1000 μg/ml**
	
B9	4.32%	34%	60.39%
HELA	11.26%	20.32%	56.74%
A549	13.41%	28.84%	36.40%
AGS	20.46%	43%	60%
JURKAT	6%	10.71%	

This assay was repeated at least three times, producing similar results.

## References

[B1] Park EJ, Pezzuto JM (2002). Botanicals in cancer chemoprevention. Cancer Metastasis Rev.

[B2] Huang KC (1999). The Pharmacology of Chinese Herbs.

[B3] Demeule M, Michaud-Levesque J, Annabi B, Gingras D, Boivin D, Jodoin J, Lamy S, Bertrand Y, Beliveau R (2002). Green tea catechins as novel antitumor and antiangiogenic compounds. Curr Med Chem Anti-Cancer Agents.

[B4] Dorai T, Aggarwal BB (2004). Role of chemopreventive agents in cancer therapy. Cancer Lett.

[B5] Ghersi D, Wilcken N, Simes RJ (2005). A systematic review of taxane-containing regimens for metastatic breast cancer. Br J Cancer.

[B6] Lopez-Lazaro M (2002). Flavonoids as anticancer agents: structure-activity relationship study. Curr Med Chem Anti-Cancer Agents.

[B7] Surh YJ (2003). Cancer chemoprevention with dietary phytochemicals. Nat Rev Cancer.

[B8] Philip PA (2005). Experience with docetaxel in the treatment of gastric cancer. Semin Oncol.

[B9] Chung KT, Wong TY, Wei CI, Huang YW, Lin Y (1998). Tannins and human health: a review. Crit Rev Food Sci Nutr.

[B10] Tang W, Hemm I, Bertram B (2003). Recent development of antitumor agents from Chinese herbal medicines; Part I. Low molecular compounds. Planta Med.

[B11] Kinghorn AD, Su BN, Jang DS, Chang LC, Lee D, Gu JQ, Carcache-Blanco EJ, Pawlus AD, Lee SK, Park EJ, Luendent M, Gills JJ, Bhat K, Park HS, Mata-Greenwoode E, Song LL, Jang M, Pezzuto JM (2004). Natural Inhibitors of carcinogenesis. Planta Med.

[B12] Akihisa T, Yasukawa K, Oinuma H, Kasahara Y, Yamanouchi S, Takido M, Kumasi K, Tamura T (1996). Triterpene alcohols from the flowers of compositae and their anti-inflammatory effects. Phytochemistry.

[B13] Della Logia R, Tubaro A, Sosa A, Becker H, Saar S, Isaac O (1994). The role of triterpenoids in the topical anti-inflammatory activity of Calendula officinalis flowers. Planta Med.

[B14] Kalvatchev Z, Walder R, Garzaro D (1997). Anti-HIV activity of extracts from Calendula officinalis flowers. Biomed Pharmacother.

[B15] Perez-Carreon JI, Cruz-Jimenez G, Licea-Vega JA, Arce-Popoca E, Fattel-Fazenda S, Villa-Trevino S (2002). Genotoxic and anti-genotoxic properties of Calendula officinalis extracts in rat liver cell cultures treated with diethylnitrosamine. Toxicol in Vitro.

[B16] Pommier P, Gomez F, Sunyach MP, D'Hombres A, Carrie C, Montbarbon X (2004). Phase III randomized trial of Calendula officinalis compared with trolamine for the prevention of acute dermatitis during irradiation for breast cancer. J Clin Oncol.

[B17] Boucaud-Maitre Y, Algernon O, Raynaud J (1988). Cytotoxic and antitumoral activity of Calendula officinalis extracts. Pharmazie.

[B18] Yoshikawa M, Murakami T, Kishi A, Kageura T, Matsuda H (2001). Medicinal flowers. III. Marigold. (1): hypoglycemic, gastric emptying inhibitory, and gastroprotective principles and new oleanane-type triterpene oligoglycosides, calendasaponins A, B, C, and D, from Egyptian Calendula officinalis. Chem Pharm Bull.

[B19] Neukirch H, D'Ambrosio M, Dall-Via J, Guerriero A (2004). Simultaneous quantitative determination of eight triterpenoid monoesters from flowers of 10 varieties of Calendula officinalis L. and characterisation of a new triterpenoid monoester. Phytochem Anal.

[B20] Robertson MJ, Cochran KJ, Cameron C, Le JM, Tantravahi R, Ritz J (1996). Characterization of a cell line, NKL, derived from an aggressive human natural killer cell leukemia. Exp Hematol.

[B21] Liebmann JE, Cook JA, Lipschultz C, Teague D, Fisher J, Mitchell JB (1993). Cytotoxic studies of paclitaxel (Taxol) in human tumour cell lines. Br J Cancer.

[B22] Singh RP, Dhanalakshmi S, Agarwal R (2002). Phytochemicals as cell cycle modulators: a less toxic approach in halting human cancers. Cell Cycle.

[B23] Lowe SW, Lin AW (2000). Apoptosis in cancer. Carcinogenesis.

[B24] Jo EH, Kim SH, Ra JC, Kim SR, Cho SD, Jung JW, Yang SR, Park JS, Hwang JW, Aruoma OI, Kim TY, Lee YS, Yang KS (2005). Chemopreventive properties of the ethanol extract of Chinese licorice (*Glycyrrhiza uralensis*) root: induction of apoptosis and G1 cell cycle arrest in MCF-7 human breast cancer cells. Cancer Lett.

[B25] Kundu T, Dey S, Roy M, Siddiqui M, Bhattachrya RK (2005). Induction of apoptosis in human leukaemia cells by black tea and its polyphenol theaflavin. Cancer Lett.

[B26] Garcia-Lora A, Pedrinaci S, Garrido F (2001). Protein-bound polysaccharide K and interleukin-2 regulate different nuclear transcription factors in the NKL human natural killer cell line. Cancer Immunol Immunother.

[B27] Kudo S, Tanaka J, Kashida H, Tamegai Y, Endo S, Yamano HO (2002). Effectiveness of immunochemotherapy with PSK, a protein-bound polysaccharide, in colorectal cancer and changes of tumor marker. Oncol Rep.

[B28] Ohwada S, Ikeya T, Yokomori T, Kusaba T, Roppongi T, Takahashi T, Nakamura S, Kakinuma S, Iwazaki S, Ishikawa H, Kawate S, Nakajima T, Morishita Y (2004). Adjuvant immunochemotherapy with oral Tegafur/Uracil plus PSK in patients with stage II or III colorectal cancer: a randomised controlled study. Br J Cancer.

[B29] Kariya Y, Inoue N, Kihara T, Fujii M (1992). Activation of human natural killer cells by the protein bound polysaccharide PSK independently of interferon and interleukin 2. Immunol Lett.

[B30] Algarra I, Collado A, Garcia-Lora A, Garrido F (1999). Differential effect of protein-bound polysaccharide (PSK) on survival of experimental murine tumors. J Exp Clin Cancer Res.

[B31] Smyth MJ, Crowe NY, Hayakawa Y, Takeda K, Yagita H, Godfrey DI (2002). NKT cells – conductors of tumor immunity?. Curr Opin Immunol.

[B32] Crowe NY, Smyth MJ, Godfrey DI (2002). A critical role for natural killer T cells in immunosurveillance of methylcholanthrene-induced sarcomas. J Exp Med.

